# SOCIAL WORKERS’ ROLE IN CULTIVATING ILLNESS UNDERSTANDING AND PROGNOSTIC AWARENESS: PERSPECTIVES FROM THE FIELD

**DOI:** 10.1093/geroni/igae098.2639

**Published:** 2024-12-31

**Authors:** Arden ODonnell, Judith Gonyea

**Affiliations:** Boston University, Boston, Massachusetts, United States; Boston University, Boston, Massachusetts, United States

## Abstract

For patients experiencing serious or life-limiting illnesses, informed decision-making depends on understanding prognosis and illness trajectory. Although physicians are viewed as the most appropriate group to share medical information, there is growing recognition that: a) cultivation of prognostic awareness occurs over time through a series of conversations in which patients continue to redefine medical treatments and broader life goals and b) other clinicians can contribute to these discussions. Using role theory, this qualitative study’s explores how the growing shift to a team approach is experienced by hospital-based physicians (MDs) and palliative social workers (PSWs),. Through semi-structured Zoom interviews with 17 matched MDs and PSWs across the U.S., we explored the degree of alignment or convergence of MDs’ and PSWs’ views on PSWs’ professional roles in prognostic conversations, including perceptions of role definitions, role sharing, role boundaries, and role conflicts. Interpretive description methodology, coupled with thematic analysis, revealed key insights into PSWs’ comfort levels in assuming roles and interventions in this prognostic awareness cultivation as well interprofessional convergence affirming PSWs’ role in a) assessing patients’ readiness for prognostic discussions, b) identifying gaps in patient understanding c) translating medical conditions into lived reality, and d) supporting emotional processing of patients and their families. Concerns about potential role conflicts emerged when PSWs were expected to either engage in or overstep into medically complex discussions. This study underscores the important role of social workers role in enhancing prognostic understanding and emphasizes the significance of role clarity, trust, and communication for effective interpersonal teams.

